# Chemical Identity of Interaction of Protein with Reactive Metabolite of Diosbulbin B In Vitro and In Vivo

**DOI:** 10.3390/toxins9080249

**Published:** 2017-08-14

**Authors:** Kai Wang, Dongju Lin, Xiucai Guo, Wenlin Huang, Jiang Zheng, Ying Peng

**Affiliations:** 1 School of Pharmacy, Shenyang Pharmaceutical University, P.O. Box 21, 103 Wenhua Road, Shenyang 110016, Liaoning, China; kaiwang_2009@163.com (K.W.); lindo.ngju@163.com (D.L.); xiucai1224@163.com (X.G.); 2 Department of Biochemistry, University of Washington, Seattle, WA 98195, USA; wenlin2@uw.edu; 3 State Key Laboratory of Functions and Applications of Medicinal Plants, Key Laboratory of Pharmaceutics of Guizhou Province, Guizhou Medical University, Guiyang 550004, Guizhou, China; 4 Wuya College of Innovation, Shenyang Pharmaceutical University, P.O. Box 21, 103 Wenhua Road, Shenyang 110016, Liaoning, China; 5 School of Chinese Materia Medica, Tianjin University of Traditional Chinese Medicine, Tianjin 300193, China

**Keywords:** diosbulbin B, hepatotoxicity, protein covalent binding, metabolic activation, bromine-based analytical technique

## Abstract

Diosbulbin B (DIOB), a hepatotoxic furan-containing compound, is a primary ingredient in *Dioscorea bulbifera* L., a common herbal medicine. Metabolic activation is required for DIOB-induced liver injury. Protein covalent binding of an electrophilic reactive intermediate of DIOB is considered to be one of the key mechanisms of cytotoxicity. A bromine-based analytical technique was developed to characterize the chemical identity of interaction of protein with reactive intermediate of DIOB. Cysteine (Cys) and lysine (Lys) residues were found to react with the reactive intermediate to form three types of protein modification, including Cys adduction, Schiff’s base, and Cys/Lys crosslink. The crosslink showed time- and dose-dependence in animals given DIOB. Ketoconazole pretreatment decreased the formation of the crosslink derived from DIOB, whereas pretreatment with dexamethasone or buthionine sulfoximine increased such protein modification. These data revealed that the levels of hepatic protein adductions were proportional to the severity of hepatotoxicity of DIOB.

## 1. Introduction

**Scheme 1:**
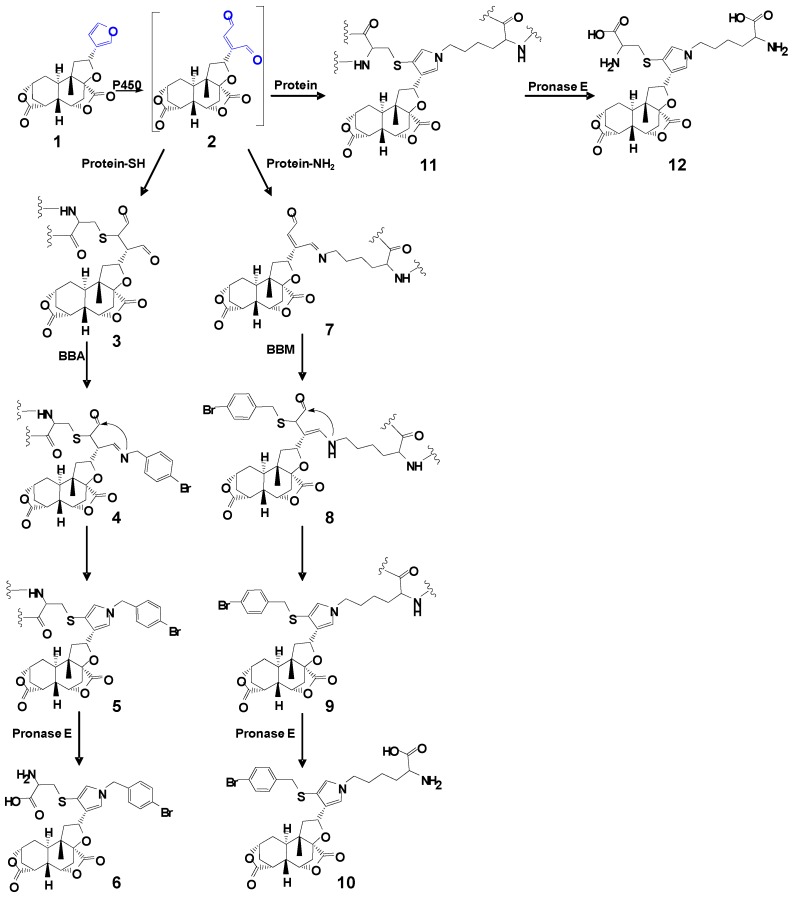
Proposed pathways of protein/amino acid residue adduct formation as a result of DIOB bioactivation.

Diosbulbin B (DIOB, **1**, Scheme 1) is a primary furanoid found in *Dioscorea bulbifera* L. (DB, Huang-Yao-Zi in Chinese) [1,2] which has been widely used in China. DB, along with its remedies, is used for the treatment of carbuncles, lung abscesses, breast lumps, and thyroid gland diseases [3,4,5,6]. In addition, the herb shows varieties of pharmacological activities, including antitumor [7], antifeedant [8], antiinflammation [9], and antisalmonellal effects [10]. Unfortunately, numerous liver injury cases have been documented to be associated with consumption of DB or remedies containing DB [11,12,13]. 

As a primary component of DB, the furanoid has been found to induce hepatic toxicity and oxidative stress in mice given (p.o.) DIOB for 12 consecutive days [14,15]. Our early study showed that administration of DIOB at 100 mg/kg produced acute liver injury by biochemical estimation of serum markers and histopathological examination. In particular, biotransformation of the furan group played an important role in DIOB hepatotoxicity [16]. Metabolic conversion of toxic furan-containing compounds to *cis*-enediones, *cis*-enedials or *γ*-ketoenals is generally suggested to be a crucial step that initiates the development of the reported toxicities [17,18,19,20]. Our preliminary study showed that DIOB was metabolized to reactive metabolite **2** (Scheme 1), an electrophilic species. P450 3A was the primary enzyme that catalyzes the metabolic activation of DIOB [21]. Toxicological studies consistently showed that pretreatment with ketoconazole (KTC, inhibitor of P450 3A) protected the animals from hepatotoxicities and hepatic GSH depletion induced by DIOB. The chemically reactive *cis*-enedial intermediate was found to react with the sulfhydryl group of glutathione (GSH) and the primary amine group of *N*-acetyl lysine (NAL) to produce a chemically stable pyrrole derivative [21,22,23]. We hypothesized that the reactive metabolite generated in situ reacts covalently with cysteine (Cys) thiol and lysine (Lys) primary amines of hepatic proteins to generate covalent binding. Protein modification has been considered to be an important mechanism of furan toxicities [24,25,26,27]. The objectives of the present study were to determine protein adduction induced by the metabolic activation of DIOB, to define the chemical identities of interactions of the reactive metabolite with proteins, and to determine whether pretreatments which effectively altered toxicity of DIOB in previous studies would also alter covalent adduct formation in a parallel fashion.

## 2. Results

### 2.1. Cys/Lys Adduction by Reactive Intermediate of DIOB

**Figure 1:**
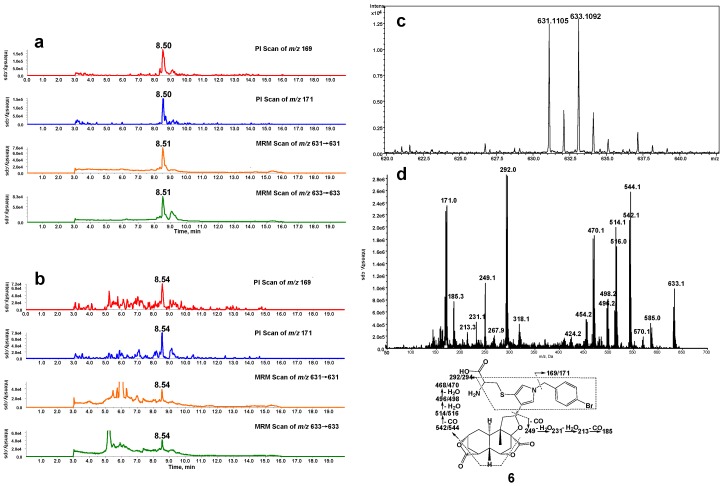
(**a**) Mass spectrometric profiles obtained from a four-channel scanning system analysis of mouse liver microsomal incubations containing DIOB, NADPH, Cys, and BBA; (**b**) Mass spectrometric profiles obtained from the four-channel scanning system analysis of mouse liver microsomal incubations containing DIOB, NADPH, and BBA, followed by exhaustive proteolytic digestion; (**c**) High resolution mass spectrum; (**d**) MS/MS spectrum of pyrrole **6**.

Microsomal incubations with DIOB supplemented with Cys and BBA produced a Cys/BBA pyrrole derivative (**6**, Scheme 1). Ions *m/z* 169 and 171 with a clear 1:1 bromine isotope ratio were considered as the characteristic fragment ions of BBA and BBM (see below), which would allow us to monitor pyrrole **6** by PI scanning of *m/z* 169 and 171. Additionally, the molecular weight of pyrrole **6** is known, enabling us to determine pyrrole **6** by MRM scanning of *m/z* 631/631 and 633/633. As expected, the four individual chromatograms demonstrated the respective peak with the same retention time (Rt = 8.5 min) (Figure 1a). The ratio in intensities of the two channels of PI scans of *m/z* 169 and 171 was around 1:1. The ratio in intensities of the detected MRM scans at *m/z* 631/631 and 633/633 was also approximately 1:1. The high resolution mass spectrum of the adduct showed the protonated molecular ions [M + H]^+^ at *m/z* 631.1105 and 633.1092 in positive mode (Figure 1c), which matches the molecular weight of elemental composition of protonated pyrrole **6** (C_29_H_32_BrN_2_O_7_S, calculated protonated molecular mass: 631.1108 and 633.1091). The ratio in intensities of the two molecular ions was around 1:1. The MS/MS spectrum obtained from MRM-EPI scanning (ion transition *m/z* 631/631 or 633/633) in positive mode elicited the indicative characteristic fragment ions at *m/z* 169/171 (loss of C_22_H_26_O_7_N_2_S), 292/294 (loss of C_16_H_21_O_7_N), 542/544 (loss of C_2_H_2_O_4_), 249 (loss of C_15_H_15_O_3_N_2_SBr) (Figure 1d).

**Figure 2:**
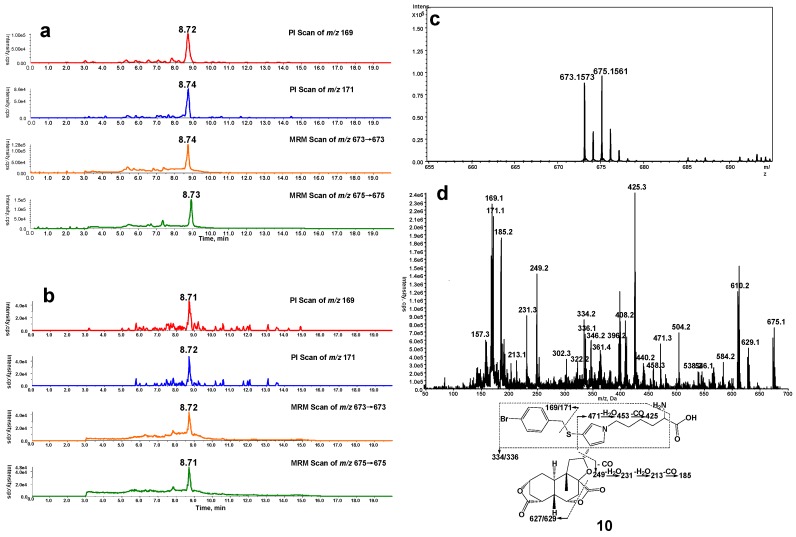
(**a**) Mass spectrometric profiles obtained from a four-channel scanning system analysis of mouse liver microsomal incubations containing DIOB, NADPH, Lys, and BBM; (**b**) Mass spectrometric profiles obtained from the four-channel scanning system analysis of mouse liver microsomal incubations containing DIOB, NADPH, and BBM, followed by exhaustive proteolytic digestion; (**c**) High resolution mass spectrum; (**d**) MS/MS spectrum of pyrrole **10**.

In a separate study, microsomes were incubated with DIOB fortified with Lys and BBM. According to the similar rationale as for the analysis of pyrrole **6**, a Lys/BBM pyrrole derivative (**10**, Scheme 1), which was monitored by a four-channel scanning system, including PI scanning of *m/z* 169 and 171 as well as MRM scanning of *m/z* 673/673 and 675/675, eluted at 8.7 min. The Lys/BBM-derived pyrrole (**10**) exhibited its protonated molecular ions [M + H]^+^ at *m/z* 673.1573 and 675.1561 with a 1:1 isotope abundance pattern, which is consistent with the elemental composition of C_32_H_38_BrN_2_O_7_S (calculated molecular mass: 673.1577 and 675.1562) (Figure 2c). The MS/MS spectrum obtained from MRM-EPI scanning (ion transition *m/z* 673/673 or 675/675) in positive mode showed the indicative fragment ions at *m/z* 169/171 (loss of C_25_H_32_O_7_N_2_S), 627/629 (loss of CH_2_O_2_), 334/336 (loss of C_16_H_21_O_7_N), 249 (loss of C_18_H_21_O_3_N_2_SBr), and 471 (loss of C_7_H_7_BrS) (Figure 2d).

Chemical syntheses of **6** and **10** were achieved by DMDO-mediated oxidation of DIOB, followed by reactions with pair Cys/BBA or Lys/BBM. The synthetic **6** and **10** revealed the same chromatographic and mass spectrometric properties as those detected in the above microsomal incubations (Supplementary Materials Figures S1 and S2).

### 2.2. Cys- and Lys-Based Protein Adduction by Reactive Metabolite of DIOB

Microsomal incubations with DIOB were performed in the presence of BBA or BBM to trap the Cys- or Lys-based protein adductions. The microsomal proteins were digested and analyzed by the four-channel monitoring systems established. As expected, pyrrole **6** or **10** was found in the corresponding microsomes supplemented with BBA or BBM, respectively (Figure 1b and Figure 2b). This suggests that Cys and Lys residues of microsomal proteins were modified by *cis*-enedial **2**. Clearly, the application of bromine-tagged reagents to trap adducted proteins resulting from metabolic activation of DIOB was successful. Not surprisingly, no such adduction was observed in the microsomes without NADPH (data not shown), implying that bioactivation was in need for the Cys- and Lys-based protein modification. To verify the formation of the Cys- and Lys-based protein adduction, we chemically oxidized DIOB with DMDO and mixed the oxidative products with microsomal protein, followed by reaction with BBA or BBM. Pyrrole derivatives were detected in the resulting reactions after complete proteolytic digestion. The synthetic pyrroles revealed the same chromatographic and mass spectral properties (Supplementary Materials Figures S3 and S4) as that of pyrroles **6** and **10** obtained from the microsomal incubations, in support of the two scenarios (Scheme 1) proposed for the interactions of Cys and Lys residues with the reactive metabolite of DIOB.

### 2.3. Cys-Lys Based Protein Crosslink Derived from Bioactivation of DIOB

**Figure 3:**
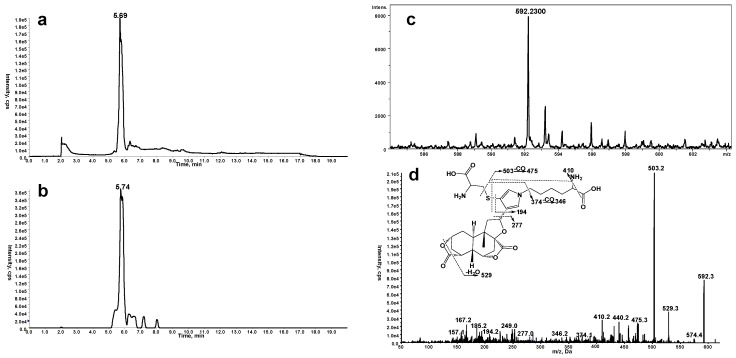
(**a**) Mass spectrometric profiles obtained from MRM scanning (ion transition *m/z* 592→503) analysis of mouse liver microsomal incubations containing DIOB, NADPH, Lys, and Cys; (**b**) Mass spectrometric profiles obtained from MRM scanning (ion transition *m/z* 592→503) analysis of mouse liver microsomal incubations containing DIOB and NADPH, followed by exhaustive proteolytic digestion; (**c**) High resolution mass spectrum; (**d**) MS/MS spectrum of pyrrole **12**.

Another scenario for protein modification derived from the reactive metabolite includes crosslink involving Cys and Lys residues (**11**, Scheme 1). The crosslink was determined by detection of pyrrole **12** (Scheme 1) in proteolytic extracts from microsomal incubations after exposure to DIOB. As an initial step, pyrrole **12** was generated by microsomal incubation of DIOB supplemented with Lys and Cys. As expected, a product responsible for pyrrole **12** (Scheme 1) was detected by MRM scanning of *m/z* 592/503 with the retention time at 5.7 min (Figure 3a). The high resolution mass spectrum of the pyrrole derivative exhibited protonated molecule ions [M + H]^+^ at *m/z* 592.2300 (Figure 3c), matching the elemental composition of C_28_H_38_N_3_O_9_S (calculated molecular mass: 592.2323). The MS/MS spectrum obtained from MRM-EPI scanning (ion transition *m/z* 592/503) in positive mode showed the indicative product ions at *m/z* 194 (loss of C_18_H_24_O_7_NS), 277 (loss of C_13_H_21_O_4_N_3_S), 374 (loss of C_9_H_18_O_4_N_2_), 410 (loss of C_4_H_10_O_4_N_2_S), 503 (loss of C_3_H_7_O_2_N), and 529 (loss of CH_3_O_3_) (Figure 3d). Pyrrole **12** was also chemically synthesized by the reaction of a mixture of Lys/Cys with DIOB after oxidation with DMDO. The synthetic pyrrole derivative demonstrated identical mass spectrometric and chromatographic identities as that of pyrrole **12** detected above (Supplementary Materials Figure S5).

A product with the same mass spectrometric and chromatographic properties as that of pyrrole **12** detected in the above study was observed in extracts of microsomes incubated with DIOB (Figure 3b), indicating the formation of Cys/Lys-based crosslink **11** (Scheme 1). To verify the crosslink, microsomal proteins were reacted with DMDO-oxidized DIOB to chemically synthesize pyrrole **11**, followed by complete proteolytic digestion. As expected, the resulting product showed the same chromatographic and mass spectrometric identities as that of pyrrole **12** formed in the microsomal reactions (Supplementary Materials Figure S6). No such pyrrole was detected in the proteolytic digestion mixture obtained frommicrosomal incubations with DIOB that were lacking NADPH (data not shown).

### 2.4. Reversibility of Protein Adduction by Reactive Metabolite of DIOB

**Figure 4:**
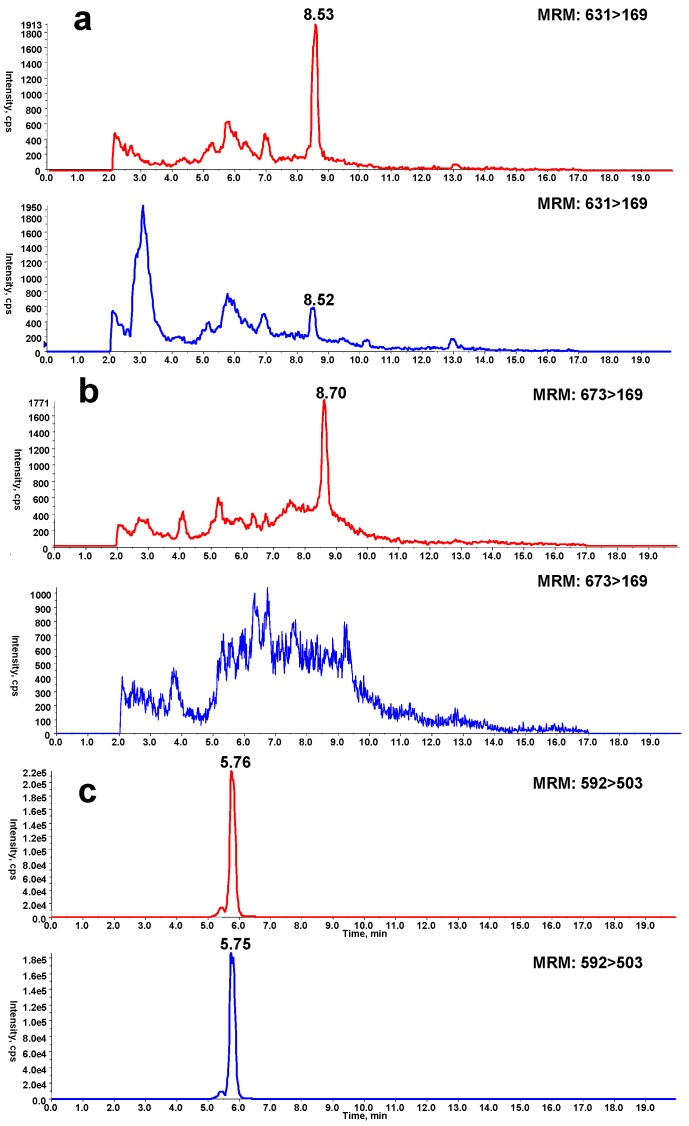
DIOB was incubated with mouse liver microsomes in the presence of NADPH, followed by reaction with BBA before (red) or after (blue) dialysis and analysis by MRM scanning (ion transition *m/z* 631→169) (**a**), followed byreaction with BBM before (red) or after (blue) dialysis and analysis by MRM scanning (ion transition *m/z* 673→169) (**b**). (**c**) Similar incubations were performedwith (blue) or without (red) dialysis before proteolytic digestion and analysis by MRM scanning (ion transition *m/z* 592→503).

To determine the reversibility of Cys or Lys residue modification by reactive intermediate, DIOB-exposed microsomes were exhaustively dialyzed and then reacted with BBA or BBM, followed by proteolytic digestion and LC-MS/MS analysis. The control samples were conducted in parallel, and the same procedure was followed except that BBA or BBM was incorporated in the microsomal mixtures before exhaustive dialysis. The levels of Cys or Lys residue modification were determined by monitoring pyrrole **6** or **10** in the digestion mixtures, respectively. A significant decrease in pyrrole **6** was observed in DIOB-exposed microsomes which had been exhaustively dialyzed before the treatment with BBA, compared with that of the corresponding control sample (Figure 4a). A similar result was obtained for the formation of pyrrole **10** detected in the digestion mixture of DIOB-exposed microsomes exhaustively dialyzed before the treatment with BBM (Figure 4b).

The reversibility of crosslink **11** was examined by comparing the generation of pyrrole **12** in the proteolytic digestion mixture of DIOB-exposed microsomes with and without dialysis. As shown in Figure 4c, little difference in the production of pyrrole **12** was observed between the proteolytic digestion mixtures from the microsomes with and without dialysis. This indicates that the first step of the reaction, either Michael addition or Schiff’s base formation (Scheme 1), was reversible, while the second step(s) of the reaction, cyclization and dehydration, was irreversible.

### 2.5. Effect of P450 3A Activity on Microsomal Protein Adduction Induced by DIOB

**Figure 5:**
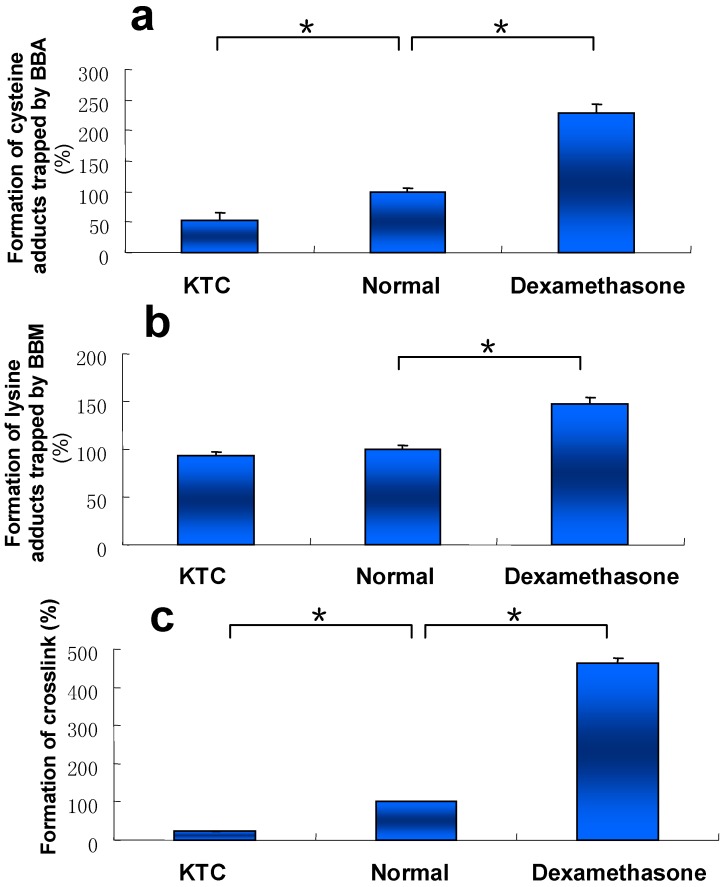
Levels of pyrrole **6** (**a**), pyrrole **10** (**b**), andpyrrole **12** (**c**) detected in microsomal incubations, containing DIOB, NADPH, and liver microsomes obtained from mice pretreated with KTC (75 mg/kg/d, 2 days), vehicle, or DEX (50 mg/kg/d, 5 days) (*n* = 3). * *P* < 0.05 were considered significantly different.

Our early work showed that metabolic activation of DIOB was mainly mediated by cytochromes P450 3A [21]. Liver microsomes with different P450 3A activity from mice pretreated with KTC (a P450 3A inhibitor), DEX (a P450 3A inducer), or vehicle (control) were individually incubated with DIOB. Once the P450 3A activity was inhibited by KTC, the formation of the three types of protein adduction was all attenuated, compared to that of DIOB-exposed microsomes from control mice (Figure 5). In contrast, elevated protein adduction was observed in DIOB-exposed microsomes from DEX-pretreated mice (Figure 5).

### 2.6. Time- and Dose-Dependent Hepatic Protein Adduction Induced by DIOB

**Figure 6:**
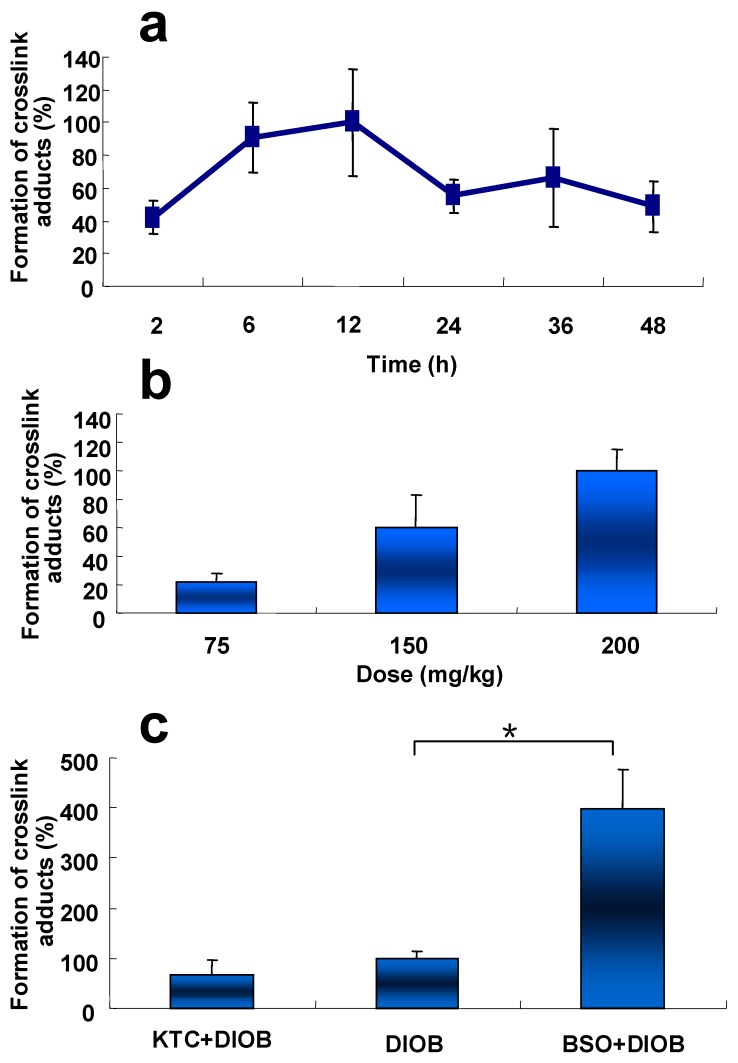
(**a**) Time-course changes in level of pyrrole **12** in mice treated with DIOB (i.p.) at 200 mg/kg^−1^ (*n* = 3); (**b**) Dose-dependent changes in level of pyrrole **12** in mice given DIOB (i.p.) at various dosages (*n* = 3); (**c**) Protective effect of KTC and synergistic effect of BSO on DIOB-induced hepatic protein modification. Mice were administered KTC (75 mg/kg/d, 2 days) + DIOB (200 mg/kg), DIOB (200 mg/kg) alone, or BSO (666 mg/kg) + DIOB (200 mg/kg) (*n* = 3). * *P* < 0.05 were considered significantly different.

Time course and dose-dependence of liver protein adduction resulting from metabolic activation of DIOB was examined in mice. The hepatic protein adduction resulting from crosslink adduction reached its peak at 12 h post treatment (Figure 6a). Additionally, the crosslink adduction increased with the increased doses of DIOB administered in animals (Figure 6b).

### 2.7. Effects of KTC and BSO on DIOB-Induced Hepatic Protein Adduction In Vivo

The formation of the protein crosslink was reduced by 10% in the liver of KTC-pretreated mice (Figure 6c), compared with that in mice administered with DIOB alone, implying that inhibition of P450 3A protected hepatic protein from DIOB-induced covalent binding. The production of the hepatic protein crosslink in mice pretreated with GSH depletor BSO was four times higher than that in animals given DIOB alone (Figure 6c). Clearly, depletion of GSH potentiated the formation of the hepatic protein modification resulting from the reactive metabolite of DIOB.

## 3. Discussion

The present study characterized the chemical identities of protein adducts resulting from the metabolic activation of DIOB both in vitro and in vivo. *cis*-Enedial **2** was identified as the reactive intermediate possibly associated with DIOB-induced hepatotoxicity [16,21]. The toxicity is likely triggered by protein covalent binding derived from the reactive metabolite. Given the structure and related chemical properties, we reasoned that the interactions of *cis*-enedial **2** with protein may occur in two ways, including modification of Cys residues by Michael addition to generate adduct **3** and reaction with Lys residues to form Schiff’s base **7** (Scheme 1). Two bromine-tagged agents, BBA and BBM, were applied to trap adducts **3** and **7**, respectively. Based upon how concentrated or diffuse the electron deficiency is, the sulfur of BBM is classified as soft nucleophile and reacts almost exclusively with the soft electrophile of *cis*-enedial **2**. The two reagents reacted with the respective adducts to produce chemically stable pyrrole derivatives **5** and **9**. Sequential proteolytic digestion of the adducts give pyrroles **6** and **10** which are derived from Cys and Lys residues, respectively. This allowed us to define the chemical identities of the interaction of protein (Cys/Lys residues) with the reactive metabolite of DIOB. The novel application of BBA and BBM as both trapping agents and labeling agents was highly selective for the detection of Cys- and Lys-based protein adduction, respectively [27]. In addition, the success in the development of the four-channel monitoring system for the detection of pyrroles **6** (*m*/*z* 169, 171, 631, and 633) and **10** (*m*/*z* 169, 171, 673, and 675) tremendously enhanced the screening selectivity and largely minimized the false positive. The bromine of the two trapping agents provided the characteristic 1:1 natural stable isotope pattern detected by mass spectrometry [27,28]. The successful application of the two bromine-tagged compounds was also particularly unique to estimate the contribution of Cys and Lys residues in the DIOB-derived protein adduction (Scheme 1).

Three types of protein modification derived from *cis*-enedial **2** were detected, including adducts **3**, **7**, and **11**. The dialysis study demonstrated that the formation of adducts **3** and **7** was reversible, while crosslink **11** was stable against exhaustive dialysis. The significance of the reversible adduction may not be as important as the crosslink in the development of DIOB toxicity. However, adducts **3** or **7** could further react with primary amines or thiols, respectively, to form the corresponding pyrrole derivatives which are not reversible any more. Thus, any interference to block the pyrrole formation would possibly slow down the development of DIOB-induced toxicity.

DIOB bioactivation-mediated microsomal protein adduction was evident. The success in the in vitro study led us to perform the protein adduction work in animals. The formation of the Cys/Lys-based crosslink (**11**, Scheme 1) in livers of mice treated with DIOB was evaluated. Our early study demonstrated that DIOB induced hepatotoxicities in a time- and dose-dependent manner in mice. The present study showed that the hepatic protein crosslink also took place in a time- and dose-dependent manner (Figure 6a,b). The adduction reached the peak at 12 h after the administration of DIOB. Our recent study demonstrated that the elevation of serum ALT reached the peak at 36 h in mice after a single dose of DIOB [16]. This indicates that a significant lag time between protein modification and the development of hepatotoxicity is required for the induction of DIOB hepatotoxicity from the onset of protein modification.

Our early work revealed that inhibition of P450 3A by KTC attenuated the production of the reactive metabolite of DIOB and protected mice from DIOB-induced hepatotoxicity [16,21]. The present study showed that pretreatment with KTC protected hepatic protein from covalent modification by the reactive metabolite of DIOB (Figure 6c), consistent with the observed reduction of protein adduction in liver microsomes from mice treated with KTC (Figure 5). The results from the protein modification and hepatotoxicity work imply a possible association between the formation of the protein adduction derived from DIOB and DIOB-induced hepatotoxicity. The dramatically increased DIOB-induced protein adduction in BSO-pretreated animals relative to that in mice given DIOB alone (Figure 6c) further suggests the correlation between hepatic protein adduction and hepatic toxicity induced by DIOB, based on our recent finding that BSO-pretreated mice were more susceptible to hepatotoxicity of DIOB [16]. It is most likely that proteins are protected against electrophilic agents and reactive oxygen species by high concentration of cellular GSH under physiologic conditions. The decreased levels of GSH caused by treatment with BSO possibly increased exposure of proteins to the reactive metabolite and thereby increased hepatic protein adduction induced by DIOB.

In conclusion, pretreatment with ketoconazole significantly decreased the formation of protein adducts, which further provided evidence for the critical role of P450 3A in metabolic activation of DIOB. The reactive intermediate of DIOB reacted with Cys and Lys residues to produce the corresponding adducts which may further form crosslink. The levels of the crosslink which may involve the critical proteins with significant importance of maintenance of cell viability correlated with the severity of hepatotoxicity induced by DIOB in mice.

## 4. Materials and Methods

### 4.1. Chemicals

DIOB was purified from DB rhizomes based on a reported protocol [29], followed by structural characterization using mass spectrometry and NMR. The purity of DIOB (λ_max_ = 210 nm) was >98% determined by an HPLC system equipped with a diode array detector. 4-Bromobenzylamine (BBA, >98%) and 4-Bromobenzylmercaptan (BBM, >98%) were purchased from Shanghai Darui Chemical Co., Ltd. (Shanghai, China) and Aladdin Industrial Co., Ltd. (Shanghai, China), respectively. Oxone, reduced nicotinamide adenine dinucleotide phosphate (NADPH), L-lysine (Lys), L-cysteine (Cys), ketoconazole (KTC, >99%), *S*-hexylglutathione (>99%), chymotrypsin, dexamethasone (DEX, >99%), DL-dithiothreitol (DTT), and buthioninesulfoximine (BSO, >99%) were obtained from Sigma-Aldrich Co. (St. Louis, MO, USA). Pronase E (>98%) was provided by Shanghai Yuanye Biological Technology Co., Ltd. (Shanghai, China). All organic solvents were of HPLC grade and supplied by Fisher Scientific (Springfield, NJ, USA).

### 4.2. Animals and Preparation of Liver Microsomes

Kunming mice (18–20 g, male) were provided by Animal Center of Shenyang Pharmaceutical University (Shenyang, China). Mice were maintained in a 25 °C room with a 12 h dark/light cycle and free access to food and water. Liver microsomes were prepared from male mice, according to our published procedure [30]. In addition, liver microsomes from mice pretreated with KTC or DEX were prepared for our study. Briefly, mice were intraperitoneally given KTC (75 mg/kg/d) for 2 successive days [31,32]. Livers were harvested at 1.5 h post the last KTC treatment for microsome preparation. In a separate study, mice were intraperitoneally administered with DEX (50 mg/kg/d) for 5 successive days, and livers were taken 24 h after the last application for the preparation of microsomes. The resulting liver microsomes were placed in a −80 °C freezer until used.

### 4.3. Reactive Metabolite Trapping

Three sets of microsomal incubations were performed as below. Set one: DIOB (200 μM) was mixed with microsomes (1.0 mg microsomal protein·mL^−1^) in 0.5 mL phosphate buffer (pH 7.4) containing MgCl_2_ (3.2 mM), and Cys (3.2 mM). Set two: similar microsomal mixture was prepared except for the replacement of Cys with Lys (10 mM) to trap the resulting reactive metabolite(s). NADPH (final concentration: 1.0 mM) were added to the two mixtures to initiate the microsomal reactions, and the resultant mixtures were placed in a 37 °C water bath with shaking for 30 min. BBA (5.0 mM) or BBM (5.0 mM) was added to the Cys- or Lys-fortified microsomes respectively, followed by additional 20 min incubation at 37 °C. Set three: microsomes (1.0 mg protein·mL^−1^) was mixed with MgCl_2_ (3.2 mM), DIOB (200 μM), Lys (10 mM), and Cys (10 mM). The reaction was initiated by addition of NADPH. The three reactions above were terminated by adding equal volumes of ice-cold acetonitrile, after 30 min incubation at 37 °C. The resulting mixtures were vortexed for 5 min. The precipitated protein was removed by centrifuging at 19,000× *g* for 10 min. The supernatants were subjected to LC-MS/MS analysis. Each microsomal incubation was conducted in duplicate. Control samples were performed without NADPH.

### 4.4. Protein Adduction and Digestion

Mouse liver microsomes (1.0 mg protein·mL^−1^) were mixed with DIOB (200 μM), followed by addition of NADPH (1.0 mM) to initiate the reactions and 30 min incubation at 37 °C. The resultant samples were mixed with BBA or BBM at a final concentration of 5.0 mM, with further incubation for 20 min. In a separate study, microsomes (1.0 mg microsomal protein/mL), DIOB (200 μM), and NADPH (1.0 mM) were mixed in 0.5 mL PBS buffer and incubated for 50 min. The resulting samples were denatured in 8.0 M urea placed in a water bath with shaking at 60 °C for 30 min, then centrifuged at 19,000× *g* for 10 min. The resultant pellets were reconstituted in 200 μL ammonium bicarbonate (50 mM, pH 8.0) containing DTT (5.0 mM) and incubated at 60 °C for 1 h. The resulting protein samples were mixed with chymotrypsin (1.0 mg·mL^−1^), Pronase E (2.0 mg·mL^−1^), and CaCl_2_ (5.0 mM ) and then were incubated at 37 °C for 15 h [27,33,34]. The digestion reactions were quenched by mixing with 0.2 mL of ice-cold methanol, vortexed, and centrifuged at 19,000× *g* for 10 min. The resulting supernatants were analyzed by the LC-MS/MS. The above experiments were conducted in duplicate. The control samples contained no NADPH. Similar procedures were applied for the protein modification study using liver microsomes obtained from mice pretreated with KTC or DEX.

### 4.5. Dialysis Study

Mouse liver microsomes (1.0 mg microsomal protein·mL^−1^) were mixed with DIOB (200 μM) in 0.5 mL phosphate buffer (pH 7.4) containing MgCl_2_ (3.2 mM). The reactions were initiated by addition of NADPH (1.0 mM). After 1 h incubation at 37 °C for 30 min, the resulting mixtures were mixed with BBA (5.0 mM) or BBM (5.0 mM) and continuously incubated for 20 min. The resulting mixtures were then dialyzed (MWCO: 3500 Da) at 4 °C for 8 h (2 × 4 h) against 2 L PBS buffer (0.1 M, pH 7.4) containing EDTA (0.5 mM). In a separate study, the incubation mixtures were dialyzed as above and mixed with BBA (5.0 mM) or BBM (5.0 mM), followed by incubation at 37 °C for 20 min. The resulting mixtures from the above two sets of experiments were denatured with urea and proteolytically digested as above. The digestion reactions were terminated by addition of 0.2 mL of methanol containing *S*-hexylglutathione as internal standard. The resultant mixtures were centrifuged at 19,000× *g* for 10 min, and the supernatants were analyzed by the LC-MS/MS system.

Another two sets of incubation mixtures were prepared by mixing the microsomes (1.0 mg microsomal protein·mL^−1^) with DIOB (200 μM) and MgCl_2_ (3.2 mM) in 0.5 mL phosphate buffer (pH 7.4). The microsomal reactions were started by addition of NADPH (1.0 mM), and the microsomal incubations were performed at 37 °C for 50 min. The resulting mixtures were divided into two parts. One was dialyzed at 4 °C for 8 h (2 × 4 h), followed by digestion, and the other was digested without dialysis. Similar procedure as described above was followed to prepare the digested samples for LC-MS/MS analysis.

### 4.6. Chemical Synthesis

To a solution of DIOB (10 mg) dissolved in 4 mL of dry ice-cold acetone was dimethyldioxirane (DMDO, 8.0 mL), pre-prepared according to a published procedure [35], dropwise added with slow stirring, followed by 10 min vigorous stirring at room temperature. The resulting mixture was left to stand for 1 h, and the solvent was evaporated under a stream of nitrogen. The residue was dissolved in 4.8 mL of PBS (pH 7.4) and divided into two fractions. One fraction was further divided into three portions, followed by addition of Cys (30 mg)/BBA(30 mg), Lys (30 mg)/BBM (30 mg), or Cys (30 mg)/Lys (30 mg), respectively. The resulting reaction mixture was stirred at 70 °C for 3 h. The other fraction was mixed with 0.6 mL mouse liver microsomes (concentration: 0.8 mg·mL^−1^) and stirred for 15 min. The resulting sample was divided into three fractions, and each fraction was individually mixed with BBA (30 mg), BBM (30 mg) and vehicle. The resulting mixtures were stirred at 70 °C for 3 h and concentrated under reduced pressure. The residues were dissolved in 50 mM ammonium bicarbonate and proteolytically digested as above. The resulting digested protein samples, along with the other three synthetic samples, were subjected to the LC-MS/MS for analysis.

### 4.7. Animal Experiments 

Kunming mice (male) were administered (i.p.) with DIOB dissolved in corn oil at 0, 75, 150, or 200 mg·kg^−1^. Livers were harvested 12 h after the administration (dose-dependent experiment) or after 2, 6, 12, 24, 36, and 48 h (time-dependent experiment conducted at 150 mg·kg^−1^).

In a separate study, mice were intraperitoneally given BSO dissolved in saline at a dose of 666 mg/kg or with vehicle. The animals were treated (i.p.) with DIOB (200 mg/kg) 1 h post dose. Another group of mice was intraperitoneally administrated with KTC (75 mg/kg/d) or vehicle for 2 consecutive days. At 1.5 h after the second KTC treatment, the mice were treated (i.p.) with DIOB at 200 mg/kg. Liver tissues were harvested 12 h post the administration.

The liver tissues (0.1 g) were homogenized in 2.0 mL phosphate buffer (pH 7.4). The resulting tissue homogenates (4 mg protein/mL) were denatured with urea and proteolytically digested as above. The digestion reactions were terminated by adding 0.2 mL of methanol containing *S*-hexylglutathione as internal standard, followed by 10 min centrifugation at 19,000× *g*. The supernatants were analyzed by the LC-MS/MS.

### 4.8. LC-MS/MS Method

The LC-MS/MS system consisted of AB SCIEX 5500 Triple Quad MS (Applied Biosystems, Foster City, CA, USA) interfaced online with an Agilent 1200 Series LC system (Applied Biosystems, Foster City, CA, USA). The separation was achieved on a Symmetry reverse-phase C18 column by Waters (4.6 × 75 mm, 3.5 μm). The mobile phases consisted of 0.1% (*v*:*v*) formic acid in acetonitrile (solvent A) and 0.1% (v:v) formic acid in water (solvent B). The gradient started at 10% A for 2 min, ramped to 90% A over 13 min, held at 90% A for 2 min, and then returned to 10% A in 1 min to equilibrate the column. The HPLC flow rate was set at 0.8 mL·min^−1^. The column temperature was maintained at 25 °C.

Cys/BBA-derived pyrrole **6** (Scheme 1) formed in vitro was analyzed by precursor ion (PI) scans of *m/z* 169 and 171 and multiple-reaction monitoring (MRM) scans of *m/z* 631/631 and 633/633 in positive mode. Lys/BBM-derived pyrrole **10** (Scheme 1) was monitored by simultaneous PI scans of *m/z* 169 and 171 and MRM scans of *m/z* 673/673 and 675/675 in positive mode. The operating parameters for the PI scans at a scan range from *m/z* 200 to 800 with a 0.5 s scan rate were set at the following values: curtain gas, 35 p.s.i.; collision gas, 7 p.s.i.; ion spray, 5500 V; temperature, 650 °C; ion source gas 1, 50 p.s.i.; ion source gas 2, 50 p.s.i.; declustering potential (DP), +70 V; entrance potential (EP), +10 V; collision energy (CE), +45 eV; and collision cell exit potential (CXP), +15 V. The operating parameters for the MRM scans as described above were similar to those for the PI scan analysis, except for CE with a value of 5 eV.

Assessment of pyrroles **6** and **10** (Scheme1) was achieved by MRM scans of *m/z* 631/169, 633/171 for **6** and *m/z* 673/169, 675/171 for **10**. The operating parameters were similar to those for the PI scan analysis. Quantitative analysis of the crosslink was achieved by MRM scan of *m/z* 592/503. *S*-Hexylglutathione (internal standard) was analyzed by MRM scan of *m/z* 392/246. Declustering potential (DP), collision energy (CE), collision cell exit potential (CXP) for pyrrole **12** responsible for the determination of the crosslink were 80, 40, 15, and those for *S*-hexylglutathione were 86, 24, 5.

The pyrroles were also analyzed on an AB SCIEX 4000 Q-Trap MS (Applied Biosystems, Foster City, CA, USA) for enhanced product ion (EPI) scans by analyzing MRM signals and to offer abundant fragmentation information for analytes. IDA was used to trigger acquisition of EPI spectra for ions exceeding 10,000 cps with exclusion of former target ions after three occurrences for 10 s. The EPI scan was run in positive mode at a scan range for product ions from *m/z* 50 to 700. The EPI scanning conditions were set as follows: scan mode, profile; step size, 0.08 Da; and scan rate, 1000 Da·s^−1^; 5.0 ms pause between mass ranges.

A microQ-TOF MS (Bruker Co., Karlsruhe, Germany) was applied to offer the accurate mass for the analytes. The Q-TOF MS system with an ESI source was operated in positive mode. The parameters of Q-TOF MS were set as follows: capillary voltage, +3800 V; nebulizer gas pressure, 1.2 bar; dry gas flow rate, 8.0 L·min^−1^; temperature, 180 °C.

## Supplementary Materials

The following are available online at www.mdpi.com/2072-6651/9/8/249/s1, Figure S1: DIOB was oxidized by DMDO, and the resulting mixture was reacted with Cys and then with BBA, followed by LC-MS/MS analysis, Figure S2: DIOB was oxidized by DMDO, and the resulting mixture was reacted with Lys and then with BBM, followed by LC-MS/MS analysis, Figure S3: DIOB was oxidized by DMDO, and the resulting mixture was reacted with microsomal protein and then with BBA, followed by complete proteolytic digestion and LC-MS/MS analysis, Figure S4: DIOB was oxidized by DMDO, and the resulting mixture was reacted with microsomal protein and then with BBM, followed by complete proteolytic digestion and LC-MS/MS analysis, Figure S5: DIOB was oxidized by DMDO, and the resulting mixture was reacted with Cys and Lys, followed by LC-MS/MS analysis, Figure S6: DIOB was oxidized by DMDO, and the resulting mixture was reacted with microsomal protein, followed by complete proteolytic digestion and LC-MS/MS analysis.

## Abbreviations

BSO	buthionine sulfoximine
BBA	4-bromobenzylamine
BBM	4-bromobenzylmercaptan
DIOB	diosbulbin B
DB	*Dioscorea bulbifera* L.
DEX	dexamethasone
CE	collision energy
HPLC/Q-TOF MS	high performance liquid chromatography/quadrupole-time-of-flight mass spectrometry
CXP	cell exit potential
DP	declustering potential
EP	entrance potential
EPI	enhanced product ion
IDA	information-dependent acquisition
KTC	ketoconazole
MRM	multiple-reaction monitoring
PI	precursor ion
