# Supplementary material for: Chemical Identity of Interaction of Protein with Reactive Metabolite of Diosbulbin B In Vitro and In Vivo
Source: Toxins (Basel). 2017 Aug 14;9(8):249. doi: 10.3390/toxins9080249 (PMC5577583; doi:10.3390/toxins9080249)
Supplement: Supplementary file 1 [file toxins-09-00249-s001.pdf]

Supplementary Materials: Chemical Identity of Interaction of Protein with Reactive Metabolite of Diosbulbin B In Vitro and In Vivo

Kai Wang, Dongju Lin, Xiucai Guo, Wenlin Huang, Ying Peng and Jiang Zheng

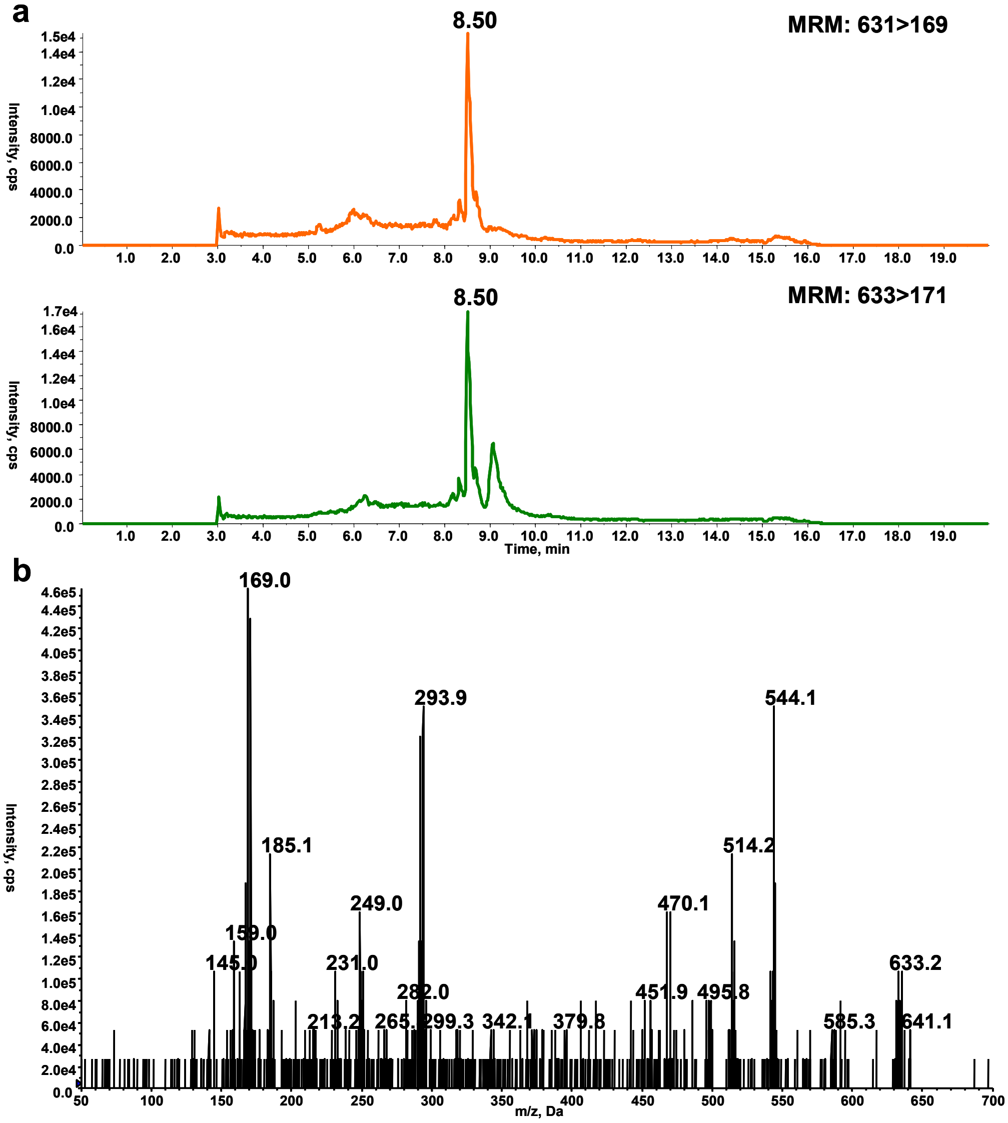

Figure S1. DIOB was oxidized by DMDO, and the resulting mixture was reacted with Cys and then with BBA, followed by LC-MS/MS analysis. **a**: Extracted ion chromatograms of MRM scans of *m*/*z* 631→169 (orange) and 633→171 (green). **b**: MS/MS spectrum of synthetic pyrrole **6.**

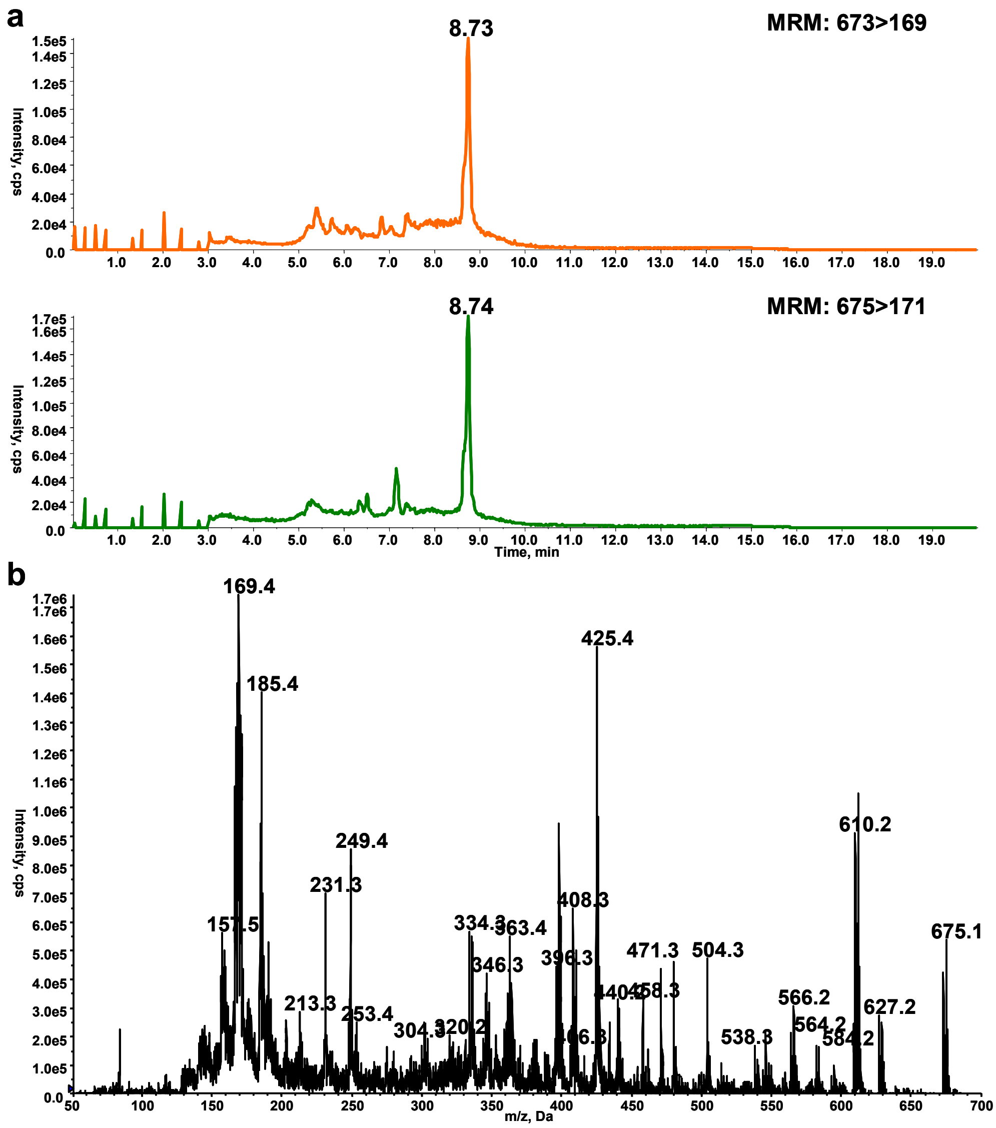

Figure S2. DIOB was oxidized by DMDO, and the resulting mixture was reacted with Lys and then with BBM, followed by LC-MS/MS analysis. **a**: Extracted ion chromatograms of MRM scans of *m*/*z* 673→169 (orange) and 675→171 (green). **b**: MS/MS spectrum of synthetic pyrrole **10.**

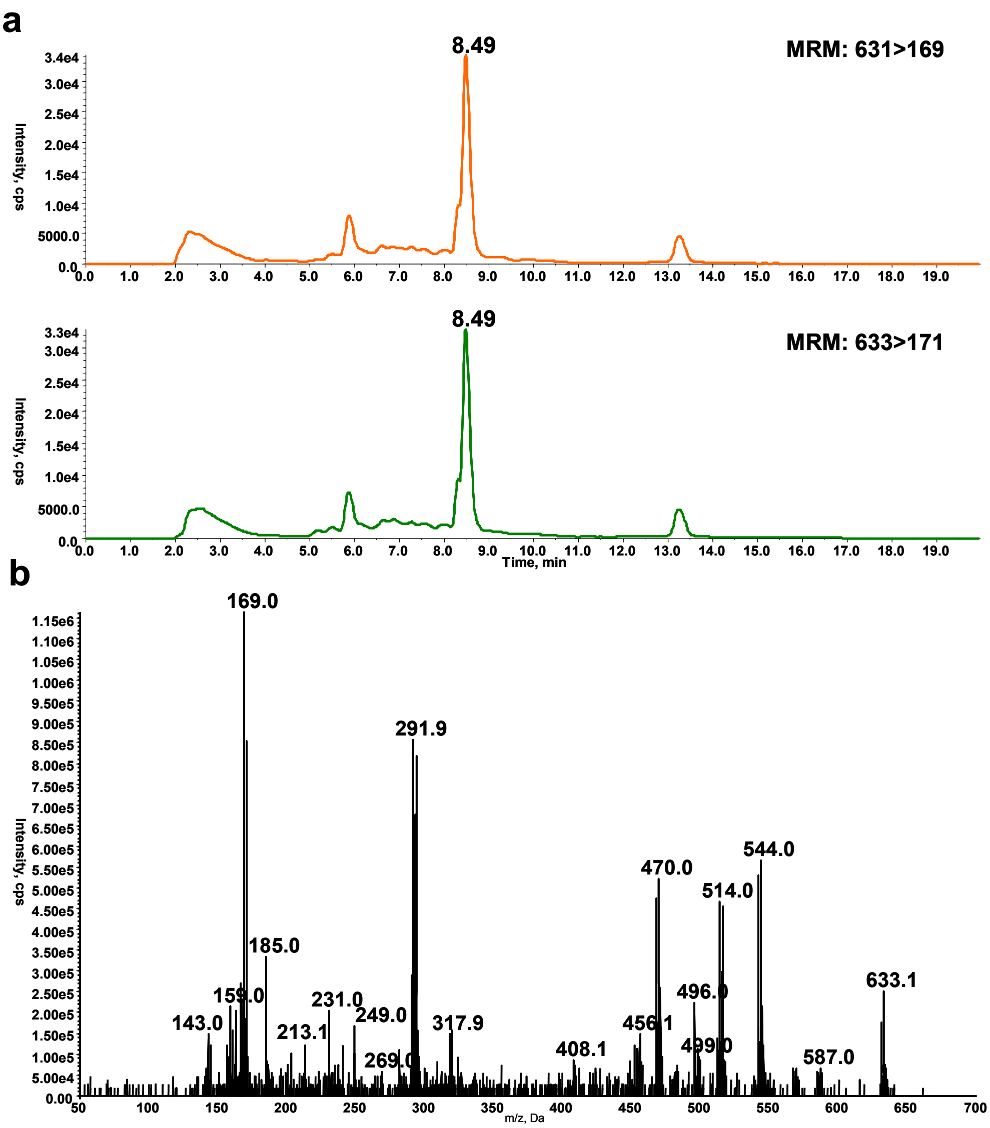

Figure S3. DIOB was oxidized by DMDO, and the resulting mixture was reacted with microsomal protein and then with BBA, followed by complete proteolytic digestion and LC-MS/MS analysis. **a**: Extracted ion chromatograms of MRM scans of *m*/*z* 631→169 (orange) and 633→171 (green). **b**: MS/MS spectrum of synthetic pyrrole **6.**

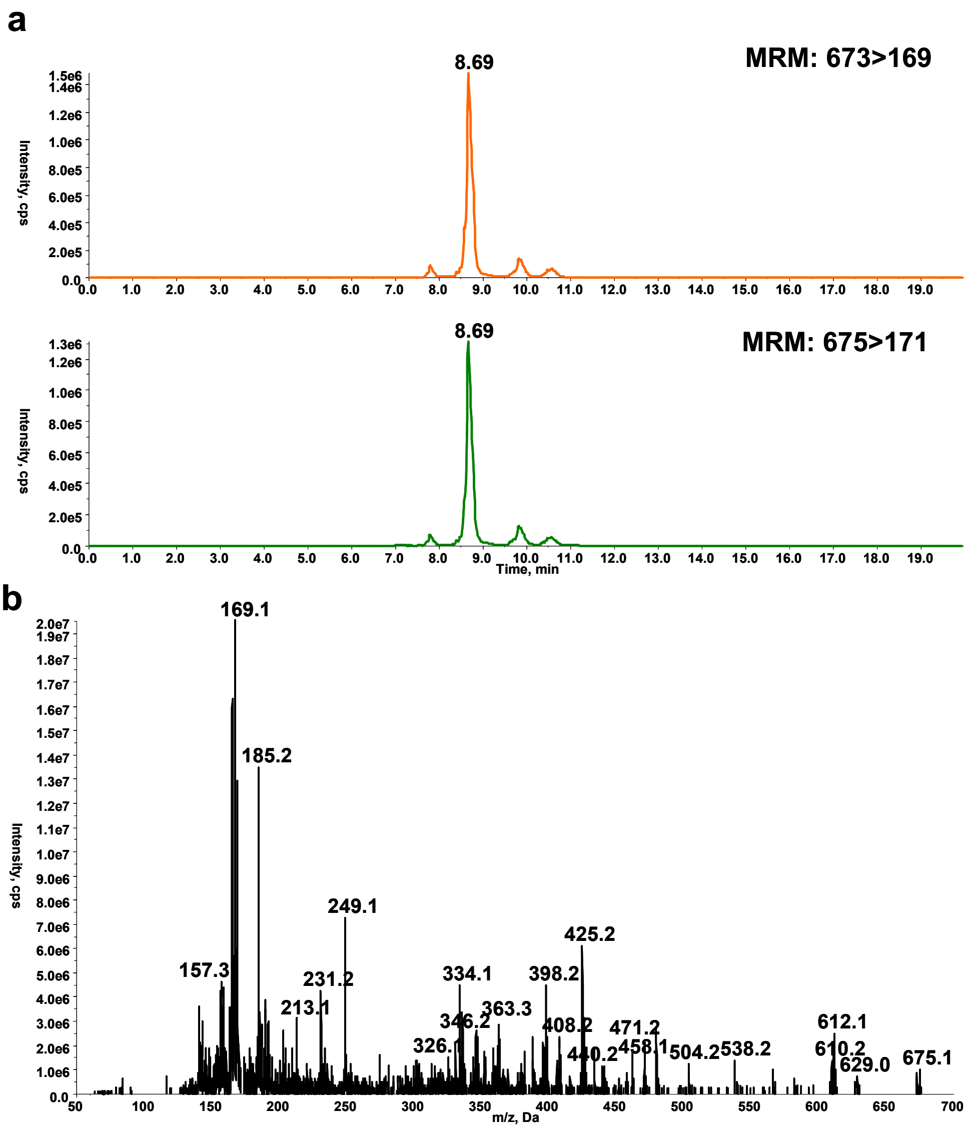

Figure S4. DIOB was oxidized by DMDO, and the resulting mixture was reacted with microsomal protein and then with BBM, followed by complete proteolytic digestion and LC-MS/MS analysis. **a**: Extracted ion chromatograms of MRM scans of *m*/*z* 673→169 (orange) and 675→171 (green). **b**: MS/MS spectrum of synthetic pyrrole **10.**

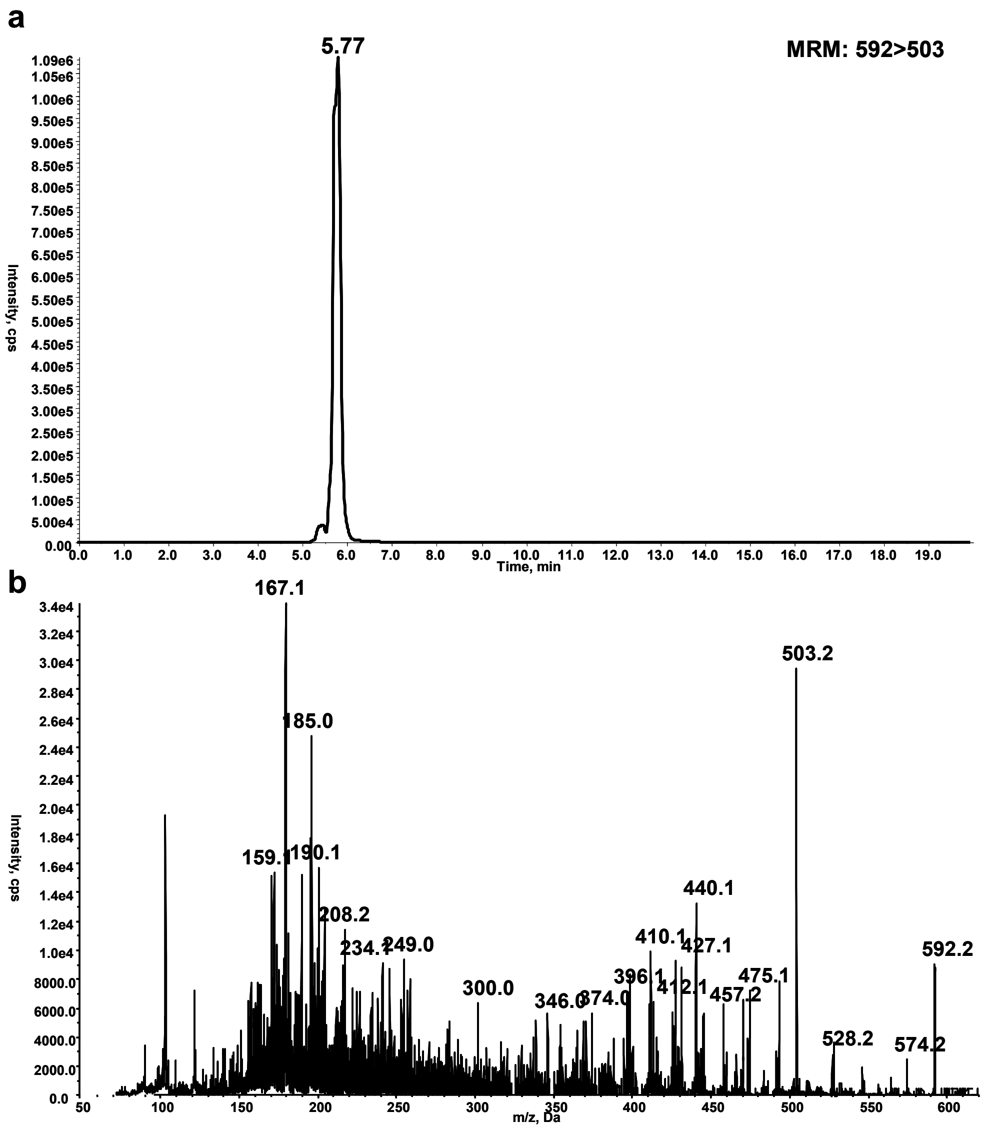

Figure S5. DIOB was oxidized by DMDO, and the resulting mixture was reacted with Cys and Lys, followed by LC-MS/MS analysis. **a**: Extracted ion chromatogram of MRM scan of *m*/*z* 592→503. **b:** MS/MS spectrum of synthetic pyrrole **12.**

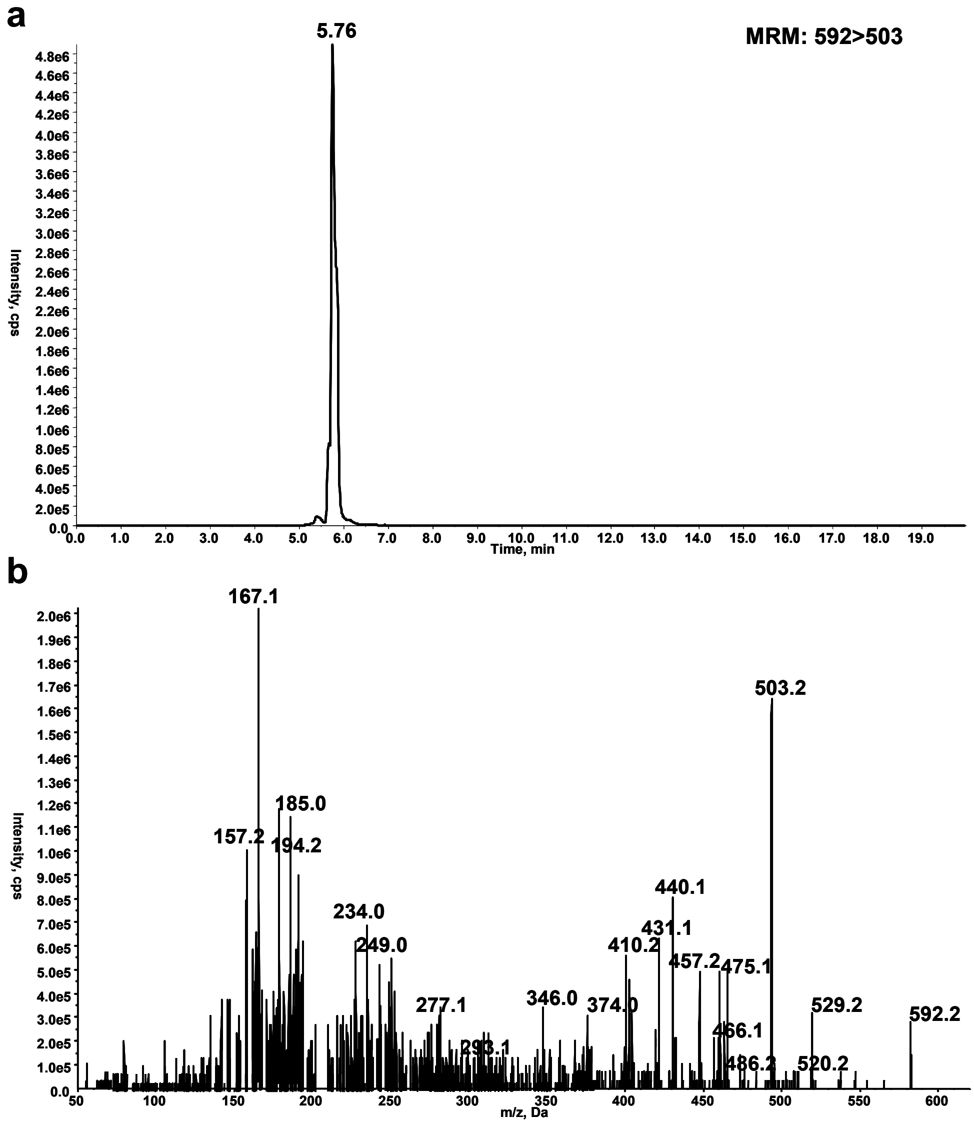

Figure S6. DIOB was oxidized by DMDO, and the resulting mixture was reacted with microsomal protein, followed by complete proteolytic digestion and LC-MS/MS analysis. **a**: Extracted ion chromatogram of MRM scan of *m*/*z* 592→503. **b**: MS/MS spectrum of synthetic pyrrole **12.**
